# Effectiveness of a Community and School-Based Intervention to Control and Prevent of Tobacco Use in Adolescents: A Field Randomized Controlled Trial

**Published:** 2019-01

**Authors:** Fariba KHAYYATI, Mohammad ASGHARI JAFARABADI, Masoud LOTFIZADEH, Asrin KARIMI, Khaled RAHMANI

**Affiliations:** 1. Social Determinants of Health Research Center, Research Institute for Health Development, Kurdistan University of Medical Sciences, Sanandaj, Iran; 2. Road Traffic Injury Research Center, Tabriz University of Medical Sciences, Tabriz, Iran; 3. Department of Statistics and Epidemiology, Faculty of Health, Tabriz University of Medical Sciences, Tabriz, Iran; 4. Social Determinants of Health Research Center, Shahrekord University of Medical Sciences, Shahrekord, Iran; 5. Neurosciences Research Center, Kurdistan University of Medical Sciences, Sanandaj, Iran

## Dear Editor-in-Chief

This article contains the authors' experience of a school and community-based intervention in Iran to prevent and reduce smoking in high school students. This has lessons for health school and community-based interventions. Adolescence is associated with several risky behaviors, such as increased use of tobacco (1). If the current trend continues, 250 million living children and adolescents who continue tobacco use into adulthood will die of health problems related to tobacco use (2). Given the global epidemiologic transition from poverty diseases to non-communicable diseases, the burden of disease and health risks among adolescents and young adults has changed significantly due to the undeniable role of substance use (3), including Iran (4). Therefore, a need for prevention and control programs of tobacco among adolescents and different implementation methods cannot be understated. Tobacco use prevention programs administered in schools are effective in reducing future smokers (5), although the interpretation of evidence for school-based prevention programs are affected by methodological issues.

We conducted a field randomized controlled trial in East Azerbaijan, Iran, during the 2014–15 school year. Study subjects (n=4422) included high school students (intervention group=1965, control group=2457). Data were collected through self-reporting questionnaires and analyzed using SPSS ver. 23 (Chicago, IL, USA).

The six-month intervention program consisted of training and environmental adaptations in cooperation with appropriate authorities. Training included teaching school staff about the health risks associated with tobacco use in adolescents and the health benefits of quitting. Physical education teachers were selected to train students about the health risks of tobacco use and how to resolve to say no to it and in 10 training sessions. In addition, students were asked to introduce their reliable peers as leader (15% of each school population) to contribute to the intervention program. The students participated in a one-hour orientation program, with question and answer time and two training videos shown in two sessions. These trained students (“peer leaders”) were requested to share their information and knowledge about tobacco use and challenge its use during the recess times. A campaign was also formed in the mosques and health centers to disseminate the message for communal effort to prevent and control tobacco access to the adolescents using leaflets and posters. Furthermore, measures were taken to ban tobacco use in public places, tobacco sale in proximity of schools, and sale to high school students. These measures were coordinated and implemented with assistance from school, trade, and police authorities. In the next step, the tobacco rehabilitation center phone number was publicized using placards in the town. The town committee on tobacco use met every two months and was updated on the progress of the program by different organizations and problems were addressed. There was no intervention in the control town (group).

Once the intervention was completed, 1885 students in the intervention city and 2305 students in the control city responded to our questionnaire. The participants were high school student, 54.7% male and 45.3% female with a mean age of 15.81 (SD=1.15). The mean of cigarette start age was 12.4 (SD=3.42) and the mean for hookah smoking start age was 13.52 (SD=2.74). The proportions of students experimented with cigarette and hookah for at least one time were 10.7% and 19.7%, respectively. Age, gender, mother’s education, and locality were used as confounding variables and were controlled.

The intervention led to an increased awareness of the side effects of tobacco (mean difference=0.36, CI.95= (0.12, 0.54)), prevention of negative changes in attitude towards tobacco (mean difference=1.59, CI.95= (−2.26, − 0.92)), and prevention of behavioral intention to tobacco use (mean difference = 0.43, CI = (0.06, 0.81), *P*<0.001). Post-intervention follow-up showed that initiating cigarette use after six months increased non-significantly in both groups but the changes within group were significant and higher in the control group. Hookah use increased significantly after six months in the control group and differed significantly from the baseline (*P*<0.02) and from the intervention group (*P*<0.001). Cigarette use increased in both groups in the past six months and 30 d but the increase in the past 30 d was higher in the intervention group (*P*<0.001). Quitting cigarette increased in the intervention group but decreased in the control group with a significant difference (*P*<0.001). In the past six months, the start of tobacco use, especially the hookah was significant in the control group, implying the need for urgent attention to smoking trend among the youth. Our results showed that intervention programs are effective in preventing tobacco use in nonsmokers than those who already smoke. Involving teachers in policies, and encouraging participation and cooperation among different authorities of community contribute to the control and prevention of tobacco use.
